# Head Acceleration Events in Male Community Rugby Players: An Observational Cohort Study across Four Playing Grades, from Under-13 to Senior Men

**DOI:** 10.1007/s40279-023-01923-z

**Published:** 2023-09-07

**Authors:** Melanie D. Bussey, Danielle Salmon, Janelle Romanchuk, Bridie Nanai, Peter Davidson, Ross Tucker, Eanna Falvey

**Affiliations:** 1 School of Physical Education, Sports and Exercise Sciences, University of Otago, Dunedin, New Zealand; 2 New Zealand Rugby, Wellington, New Zealand; 3 Institute of Sport and Exercise Medicine, University of Stellenbosch, Stellenbosch, South Africa; 4 World Rugby, Dublin, Ireland; 5 School of Medicine & Health, University College Cork, Cork, Ireland

## Abstract

**Objectives:**

The aim of this study was to examine the cumulative head acceleration event (HAE) exposure in male rugby players from the Under-13 (U13) to senior club level over 4 weeks of matches and training during the 2021 community rugby season.

**Methods:**

This prospective, observational cohort study involved 328 male rugby players. Players were representative of four playing grades: U13 (*N* = 60, age 12.5 ± 0.6 years), U15 (*N* = 100, age 14.8 ± 0.9 years), U19 (*N* = 78, age 16.9 ± 0.7 years) and Premier senior men (*N* = 97, age 22.5 ± 3.1 years). HAE exposure was tracked across 48 matches and 113 training sessions. HAEs were recorded using boil-and-bite instrumented mouthguards (iMGs). The study assessed the incidence and prevalence of HAEs by ages, playing positions, and session types (match or training).

**Results:**

For all age grades, weekly match HAE incidence was highest at lower magnitudes (10–29 g). Proportionally, younger players experienced higher weekly incidence rates during training. The U19 players had 1.36 times the risk of high-magnitude (> 30 g) events during matches, while the U13 players had the lowest risk compared with all other grades. Tackles and rucks accounted for the largest HAE burden during matches, with forwards having 1.67 times the risk of > 30 g HAEs in rucks compared with backs.

**Conclusions:**

This study provides novel data on head accelerations during rugby matches and training. The findings have important implications for identifying populations at greatest risk of high cumulative and acute head acceleration. Findings may guide training load management and teaching of skill execution in high-risk activities, particularly for younger players who may be exposed to proportionally more contact during training and for older players during matches.

**Supplementary Information:**

The online version contains supplementary material available at 10.1007/s40279-023-01923-z.

## Key Points

**Table:** 

Weekly exposure: For the highest age grade, matches accounted for 65% of the total weekly head acceleration events (HAEs); however, match contribution decreases steadily with age such that it was 56% in U19, 44% in U15 and just 41% in U13, likely reflecting the difference in match intensity with playing age and maturity.
HAE incidence: For all ages, HAEs of 10–29 g had the highest incidence rates (5–12 per player hour), whereas high magnitude (> 30 g) HAEs were rare (1–2 per week). For Premier and U19 players, > 30-g events were three times more likely to occur during matches rather than trainings.
Playing age: Compared with other grades, U19 players had the highest prevalence (> 60%) of high-magnitude (> 30 g) HAEs. This was attributed to the intensity of matches in the U19 ‘1st XV’ squads. Conversely, < 30% of U13 players experienced high-magnitude HAEs during match play.
HAEs by position: The weekly HAE incidence rate was higher for forwards compared with backs. The positional difference was largest at low magnitudes in older players for match exposure, with younger players (U13) showing no significant difference between positions.
Tackles and rucks: These accounted for over 80% of HAEs during matches. Forwards had a higher prevalence of > 30-g events in rucks but there were no positional differences in the tackle-related > 30-g events.

## Introduction

With over 9 million registered players in more than 120 countries, rugby is a popular and growing contact sport [1]. Growth has been attributed to the sport's increased media coverage, improved safety regulations, and development of community youth programmes [2]. In New Zealand, a country with a population of five million, the significant participation of 155,863 players in 2022 highlights the sport's popularity at the community level (NZR 2022 Player Database). While the growth of rugby at the youth and community levels presents an exciting opportunity for participation and engagement, addressing the potential safety concerns associated with the sport is crucial, and currently literature on the youth and community game is lacking.

The frequency of contact phases in rugby, such as tackles, rucks, scrums, mauls and lineouts, raises player safety concerns [3–5]. These phases often involve intense competition for ball possession, which can lead to a higher likelihood of unintentional head impact events and subsequently increase the risk of concussions for rugby players [3, 6]. In order to enhance player safety, it is essential to gain a comprehensive understanding of the contextual factors contributing to acute head injuries during contact phases [3]. On the other hand, recent insights have highlighted the potential influence of contact frequency on the brain injury threshold for contact sport athletes [7]. As a result, there is a growing emphasis on monitoring the severity of head impact events, especially those that do not immediately result in clinical outcomes, as a crucial aspect of player welfare strategies [8–11].

Head impact severity can be objectively described using linear and angular head kinematics to derive kinematic-based injury criteria or can serve as an input to finite element models that estimate the strain-based response of the brain tissue as a result of the impact event [12–14]. However, until recent advances in instrumented mouthguards (iMG), there have been limitations in the accuracy of on-field monitoring of head kinematics [15–17]. iMG technology provides a significant opportunity to monitor head kinematics because, in many countries, community rugby players are required or at least highly encouraged to wear mouthguards [18, 19]. In addition, iMG head kinematics can be obtained with greater accuracy compared with skin or helmet-mounted sensors, owing to improvements in the coupling of instrumentation to the athlete's skull via the dentition [15, 20]. This advancement allows practitioners and researchers to build longitudinal datasets of cumulative head acceleration event (HAE) exposures, providing critical insights into the potential relationships between day-to-day rugby head kinematic load and injury aetiology.

There has been little investigation into the frequency, magnitude, and distribution of HAEs sustained by junior and amateur male rugby players [8, 21]. Furthermore, no studies have investigated exposure to HAEs during training and no published studies have reported head kinematics in Under-13 (U13) to senior male community rugby grades. Therefore, this study was conducted to document the frequency and magnitude of HAEs in male rugby players from the U13 to Senior Premier club levels over 4 weeks of matches and training during the 2021 community rugby season. This study aimed to describe the HAE burden at different age levels and playing positions during match play and training.

In this manuscript, the authors focus on presenting data from the male participants in the ORCHID study, which is a larger research project investigating head acceleration events in over 700 community rugby players. The decision to subset the total project by sex was made due to substantial differences in exposure between male and female rugby players. By focusing solely on the male participants, the authors aim to provide a more detailed examination of head acceleration events within this particular subgroup of community rugby players. This approach allows analyses of the data in the appropriate exposure context, considering the unique characteristics and experiences of male rugby players, with a focus on playing grade/age.

## Methods

### Participants

This prospective observational cohort study examined HAE exposure by using iMGs in 328 male rugby players across 48 matches and 113 training sessions during the 2021 community rugby season. Participants were recruited from local clubs and schools, and represented the spectrum of community rugby where contact was permitted. Consenting players ranged in age from 10 to 30 years across the four playing grades (U13, U15, U19, and Senior Premier men). Parental/guardian assent was obtained for players younger than 16 years. The U13 and Premier grades comprised community-based club teams while the U15 and U19 grades comprised school-based teams. All the U19 teams, except one, were first-fifteens for their respective schools, which is the top-tier competition for secondary schools. The Premier (Prem) teams are considered the highest level of senior men’s (i.e., adult) amateur community rugby in NZ. All teams were recruited from the Dunedin metropolitan area of New Zealand and were members of the Otago Rugby Union. The population was representative of the cultural diversity within New Zealand, 58% (*n* = 186) identified as NZ European, 17% (*n* = 55) as Māori, 22% (*n* = 68) as Pasifika and 3% (*n* = 11) as other. Pasifika is a broad and diverse term that encompasses individuals from, or whose ethnic heritage links them to various Island nations and communities (e.g., Samoa, Tonga) in the South Pacific [22]. A detailed demographic breakdown is provided in Table 1. The study was conducted in accordance with the ethical principles outlined in the Declaration of Helsinki, and ethical approval was granted by the university human ethics committee (approval number H21/056).

**Table 1:** Participant demographic details for all players (*n* = 328)

Grade	Matches/trainings	Mean player mins/match Mean (SD)	Mean player mins/training Mean (SD)	Position	*N*	Age (y)		Height (cm)		Weight (kgs)		Rugby experience (y)	
						Mean	SD	Mean	SD	Mean	SD	Mean	SD
U13	10/32	54.1 (8.6)	64.3 (19.3)	Forwards	28	12.4	0.3	161.8	7.9	63.0	15.9	6.0	2.6
				Backs	32	12.4	0.6	159.0	8.3	48.4	7.7	6.6	2.4
U15	14/23	61.9 (16.2)	70.8 (19.6)	Forwards	56	14.8	0.6	178.7	7.0	85.1	18.1	8.1	3.2
				Backs	44	14.9	0.7	175.8	5.8	69.8	10.4	9.0	2.9
U19	13/29	59.3 (20.3)	63.6 (17.3)	Forwards	43	16.7	1.1	182.4	6.0	98.7	16.0	9.9	2.7
				Backs	35	17.0	0.6	178.6	6.2	79.3	9.4	10.0	3.7
Premier	11/29	68.5 (24.6)	70.6 (11.6)	Forwards	52	23.1	3.4	185.4	6.0	107.5	11.6	15.2	4.8
				Backs	45	22.1	2.7	181.8	5.3	89.0	8.1	14.7	3.5

The total sample population was *n* = 328. Fifteen players played across both the U15 and U19 grades, their demographic data is included in each cohort. One player in the U15 grade played in two teams; his demographic details are only included once in the U15 cohort. Seven players did not report their playing position (U13 *n* = 5; U15 *n* = 2); their data is not reported in the table

### Study Equipment

A qualified dentist fitted each athlete with a boil-and-bite iMG (Prevent Biometrics^®^, MN, USA) to maximise coupling to the dentition of the player. The iMG has an embedded proximity sensor that detects the coupling quality with the dentition and tracks the 'on-tooth' time. It is further equipped with a 3.2 kHz triaxial accelerometer and gyroscope to capture linear and angular kinematics and has been validated in the laboratory and field [16, 17, 23]. The precision of the mouthguard in detecting true on-field impact events in male professional rugby league players was reported at 89% (CI 87–92) [16].

All rugby sessions were video recorded with two high-definition cameras from the side-on and end-on field angles. For matches, the referees wore a head-mounted GoPro (Hero8, GoPro Inc., USA) at a third angle. The camera video footage was synced and imported into Hudl Sportscode (v 11, Agile Sports Technologies Inc., NB, USA), along with an XML file containing all iMG event data for the respective match or training. The iMG trigger threshold was set at 5 g on a single axis, with a 50-ms sampling window [24]. The iMG data were then time synchronised to the Sportscode timeline using the time flashes captured in the video. The unique serial number for each iMG was then matched to the corresponding player’s jersey number.

**Fig. 1:**
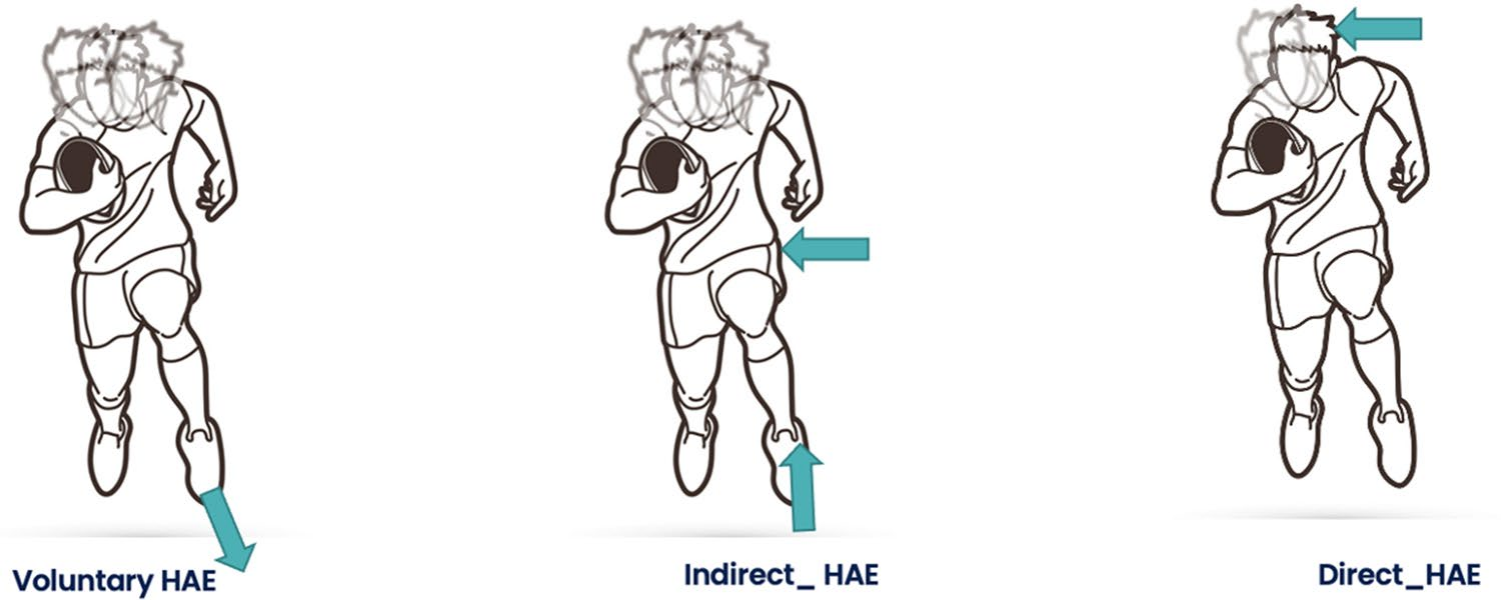
Schematic of the three types of head acceleration events (HAEs): voluntary, indirect and direct (L–R). A voluntary HAE (left) is generated through an individual’s self-acceleration or deceleration events such a running, changing direction or jumping. An indirect HAE (middle) is generated when contact is made with the player’s body resulting in an inertial loading of the head. Lastly, a direct HAE (right) is caused by direct contact with the head

HAEs were video verified as direct, indirect, or voluntary events (Fig. 1) by a trained analyst and confirmed by a second reviewer [25]. Events were labelled as ‘unclear’ when the reviewer could not confirm the HAE mechanism because the event occurred off-camera or was obscured from camera view. As per CHAMP recommendations, all raw acceleration waveforms associated with verified and unclear events were inspected for signal quality prior to inclusion in the final dataset [26].

While all match data were video verified, it was impossible to video verify all training data. This was due to a range of logistical issues around training sessions such as poor pitch lighting at night, fewer camera angles and player adherence to bib/jersey numbers. Therefore, temporospatial data windowing was performed to identify HAEs. Temporospatial windowing involves the temporal alignment of proximity data from the iMG, with temporal timestamps of HAEs and the temporal window of block-coded training events (Supplementary Fig. 1, see electronic supplementary material [ESM]) [27].

### Data Reduction and Processing

All post-processing and data reduction were performed using purpose-written MATLAB routines (R2021b, MathWorks Inc., California, USA). The raw linear acceleration and angular velocity data from the iMG accelerometer and gyroscope were imported into MATLAB and filtered using a 200 Hz low-pass fourth-order Butterworth filter [28]. The filtered data were then transformed into the head centre of mass (COM). The location and coordinate system for the head COM were defined as either the 50th or 5th centile male based on the Hybrid III crash test anatomical model [29, 30]. The 5th centile was used for athletes who fit within the 5th centile of the population by height and weight. All other athletes were treated as 50th centiles.

### Statistical Analysis

Incidence was calculated for each player based on the exposure type and time normalised to 60 min (E1). Player exposure time (in minutes) was extracted from the video footage for each instrumented player. Weekly incidence was calculated as HAE exposure over three units (two trainings and one match) of player exposure (E2). The relative risk that a player would experience a high-magnitude HAE was calculated and compared between grades and playing positions (RR, E3) [31]. The threshold for high-magnitude HAE was taken as the cohort median peak linear acceleration (PLA) plus interquartile range (IQR) (i.e., 75th centile) for direct HAEs (Supplementary Table 1, see ESM), based on the rationale that direct contact mechanisms carry a higher risk of injury.

1 $$\mathrm{Player\,IR}_{60}=\left(\frac{\sum HAE\, per\, threshold\, band }{\sum Playing\,\mathrm{min}\frac{}{60}}\right)$$

2 $$\mathrm{Player\,IR_{week}}=\left[\frac{\sum HAE\, per\, threshold \,band }{\sum Playing\,\mathrm{min }/60}\right]\times \left(3/week\right)$$

3 $$RR= \frac{A/(A+B)}{C/(C+D)}=\frac{Age\, group\, {HAE}_{high}/(Age \,group\, {HAE}_{high}+ age\, group\, no\, {HAE}_{high} )}{All\, other \,age \,{HAE}_{high}/(All \,other\, age\, {HAE}_{high}+All\, other\, age\, no\, {HAE}_{high})}$$

Demographic details, including age, height, weight, and years of rugby experience, are reported using the mean and standard deviation (SD). Statistical analyses were conducted using R (v 4.0.3; R_Core_Team 2015) and MATLAB (alpha *p* ≤ 0.05). Inter-rater agreement was evaluated for verifying HAE events as either Yes/No or event type Direct/Indirect with Cohen's kappa. Owing to evidence of overdispersion from residual analyses, negative binomial regression was used to model the counts of impacts at or above each threshold from 5 to 40 g for linear acceleration (in 1-g increments), with log-time (match or training) included as an offset. Position (forward or back), grade (U13, U15, U19, or Prem), and session type (match or training) were included, along with three- and two-way interactions. Crossed random effects accommodated repeated measures for the players, teams, and matches.

## Results

Over the 2021 season, 328 male players across the four grades wore iMGs during a combined total of 48 matches and 113 training sessions. Thirteen players played in both U15 and U19 grades, leading to a total sample of 341 player matches (U13, *n* = 60; U15, *n* = 100; U19, *n* = 78; Prem, *n* = 97). A total of 17,865 HAEs were captured throughout the study. Of the total events, 1820 were removed, 95 contained errors indicative of sensor malfunction (Supplementary Fig. 3, see ESM), and 1725 were < 5 g, leaving the remaining 16,450 events for the analysis. The inter-rater agreement for verification of HAEs as 'yes/no' was *κ* = 0.873 (95% CI 0.824–0.923) and as 'direct/indirect' was *κ* = 0.826 (95% CI 0.781–0.870). The cohort median PLA for direct events was 26.3 g (SD 0.5), which was rounded to 30 g for RR analysis.

### Weekly Exposure

**Fig. 2:**
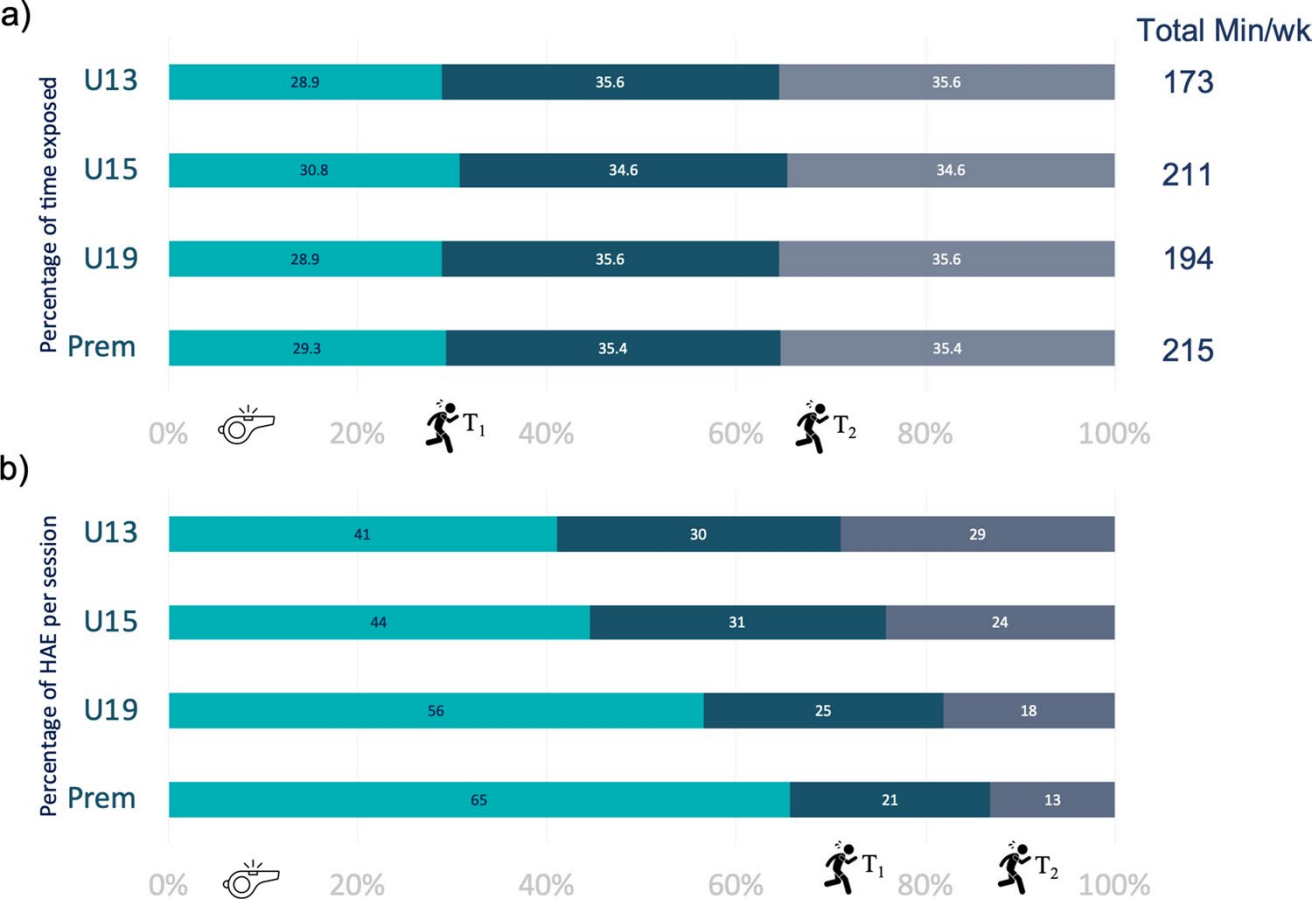
Snapshot of weekly rugby exposure per playing grade by **a** time exposure and **b** head acceleration event (HAE) exposure. A typical week was defined by one match (*turquoise*) and two trainings—T_1_ (*teal*) and T_2_ (*grey*). *Prem* Premier, *U13* Under-13, *U15* Under-15, *U19* Under-19

A typical week for the included community rugby players consisted of two training sessions and a weekend match. As shown in Fig. 2a, the training-time to match-time ratio was similar across all playing grades, with approximately 30% of the total exposure occurring during the match and 70% during the two training sessions. The proportion of HAE counts between training and matches differed between the grades (Fig. 2b). Matches accounted for 65% of the HAEs in Prem, with significantly smaller proportions of HAEs occurring during matches as the age/grade decreased (Fig. 2b).

**Fig. 3:**
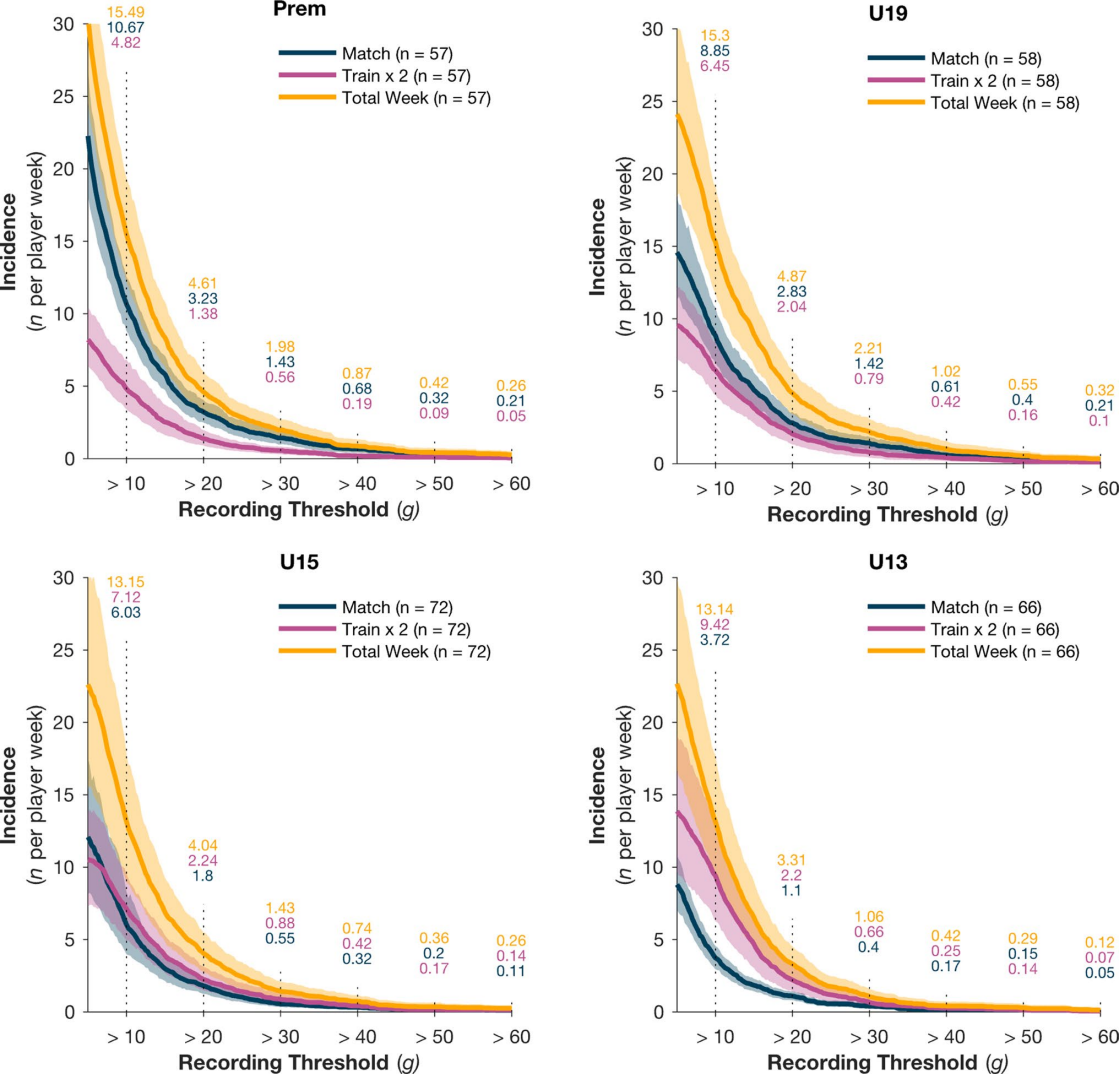
Cumulative head acceleration event (HAE) incidence rate per player week for one match (*blue*), two training sessions (*purple*) and combined (*yellow*) across four playing grades: Premier (Prem), Under-19 (U19), U15 and U13 (**a**–**d**, respectively). The *solid line* represents the mean incidence rate and *shading* represents the 95% confidence interval. HAE incident rates per peak linear acceleration magnitude band (10–60 g) are indicated by the numbers above each vertical line

The cumulative weekly incidence rates (IR) by session type are presented in Fig. 3a–d. The general incidence pattern of HAE exposure was similar for all grades. HAE incidence was significantly higher at lower magnitudes. For example, IR 10–29 g was 6–8 times higher than IR 30–60 g (Table 2). Additionally, a significant interaction (*χ*^2^ = 14.24, *p* = 0.003) between grade and session type was observed at lower HAE magnitudes, and became less evident at higher magnitudes (> 30 g) (Fig. 3a–d). The Prem group had a higher incidence of HAE exposure during matches than during training (*z* = 3.796, *p* = 0.003), with the opposite pattern observed in the U13s, who had a higher incidence of HAEs during training sessions (*z* = 3.404, *p* = 0.019).

**Table 2:** Incident rates, player prevalence and relative risk ratios for HAEs in matches and trainings across the four player grades

Player grade	Match events						Training events					
	Weekly incidence rate		Prevalence	Relative risk (RR) 30–60 g			Weekly incidence rate		Prevalence	Relative risk (RR) 30–60 g		
	10–29 g	30–60 g	30–60 g (%)	RR	95% CI	*p*-Value	10–29 g	30–60 g	30–60 g (%)	RR	95% CI	*p*-Value
U13	3.32	0.40	**28**	**0.56**	**0.38–0.83**	**0.003**	8.76	0.66	36	0.86	0.33–1.38	0.547
U15	5.48	0.55	47	0.99	0.77–1.28	0.971	6.24	0.88	42	1.39	0.89–2.06	0.155
U19	7.43	1.42	**63**	**1.36**	**1.08–1.70**	**0.008**	5.66	0.79	35	0.83	0.49–1.36	0.447
Premier	9.24	1.43	**58**	**1.19**	**0.92–1.53**	**0.178**	4.26	0.56	41	1.02	0.64–1.63	0.917

Significant values are indicated in bold

It is noteworthy that in the older grades, the 30–60 g IR for two training exposures was 0.4–0.5 times that of the single match exposure (Table 2). An adult player may experience approximately two 30–60 g events per week, but the likelihood of having such an event during training is low (Fig. 3). We also note that the youngest grades had the lowest 30–60 g IR, while U19 and Prem were similar. The prevalence of 30–60 g was highest in U19 during the match sessions, and the RR indicates their 30–60 g exposure was 1.36 times that of the rest of the population (Table 2). U13 players were least likely to experience high magnitude HAEs during matches (RR 0.56, 95% CI 0.58–0.83).

### Exposure by Playing Position

**Fig. 4:**
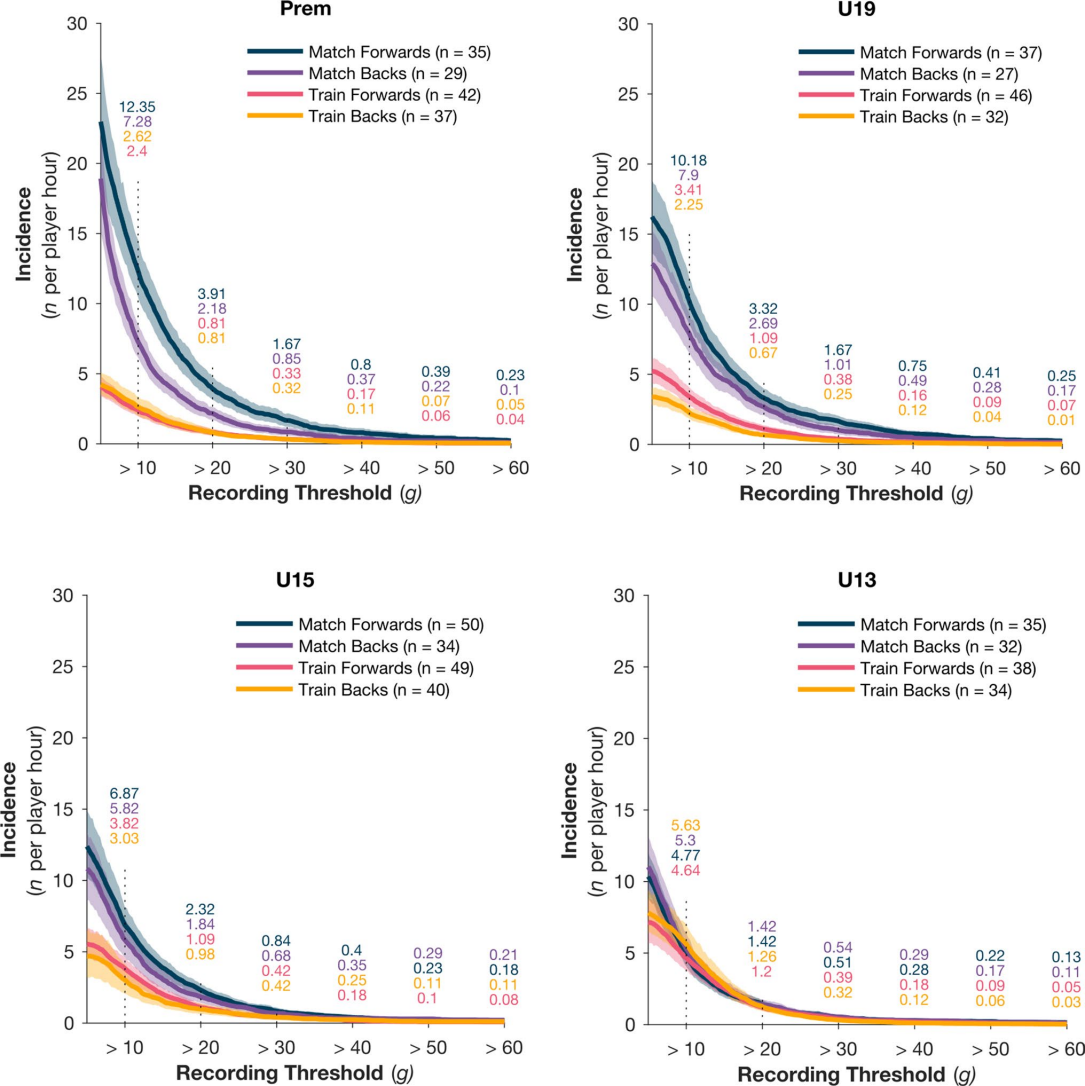
Head acceleration event (HAE) incidence rate per player hour for match forwards (*blue*), match backs (*purple*), training forwards (*pink*) and training backs (*yellow*) across the four playing grades: Premier (Prem), Under-19 (U19), U15 and U13 (**a**–**d**, respectively). The *solid line* represents the mean incidence rate and *shading* represents the 95% confidence interval. HAE incident rates per peak linear acceleration magnitude band (10–60 g) are indicated by the numbers above each vertical line

Figure 4a–d show the per-hour IR comparisons for forwards and backs during the training and match sessions. There was evidence of significant three-way interactions (*X*^2^ = 729.1, *p* < 0.001) at lower magnitudes, the evidence and magnitude of this effect diminished when higher magnitudes (> 30 g) were used. This interaction indicates that the relationship between positions and sessions changes with playing age. For *higher grades*, *forwards* experienced *higher* IR in *match* sessions. The difference between positions and sessions diminished as age decreased. For the *youngest grade* and at *lower magnitudes,* the relationship between position and sessions is opposite to the Prem men, with *backs* having the *highest IR* (> 10 g) in *training* sessions. The IR became more similar for all combinations of position, grade, and session type as HAE magnitude increased. The risk ratio further indicated no significant difference in risk associated with playing position in either match (RR 1.139, CI 0.979–1.326; *p* = 0.0916) or training (RR 0.928, CI 0.786–1.095; *p* = 0.3775). Data pertaining to the angular acceleration can be found in the ESM (Supplementary Fig. 2).

### Exposure by Contact Type

**Fig. 5:**
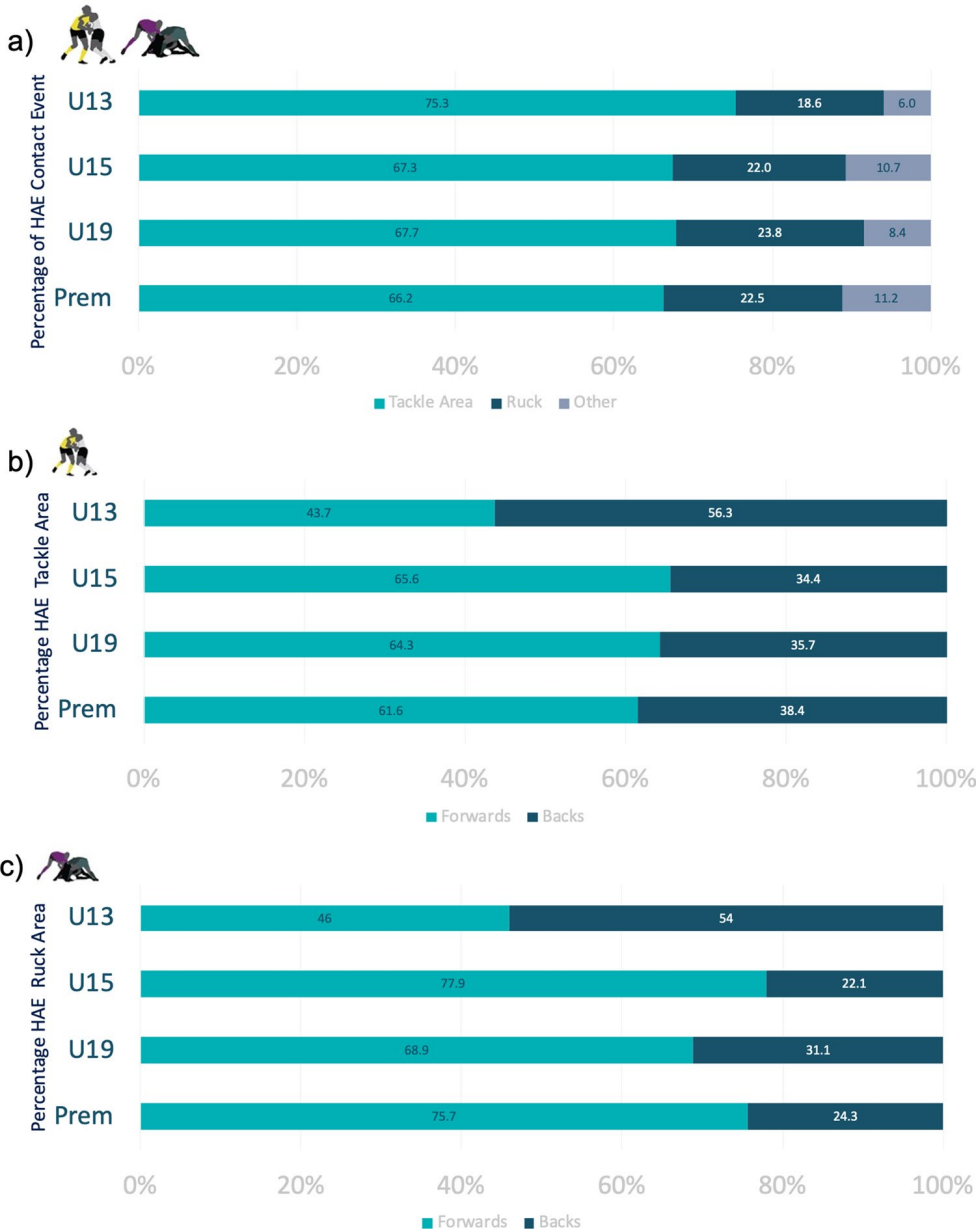
Percentage of total head acceleration events (HAEs) by playing grade for **a** all match events, **b** tackle area and **c** rucks. The tackle area includes both the tackler and the ball carrier. Rucks were defined as a phase of play with one player on the ground and at least two opposing players engaged over them. Other events include scrum, lineouts, mauls and general play. Figs b and c compare forwards and backs. *Prem* Premier, *U13* Under-13, *U15* Under-15, *U19* Under-19

During matches, most HAEs occurred during tackles and rucks, with tackles accounting for more than 60% of HAEs per grade (Fig. 5a). The rotational acceleration of the head was similar across impact events for a given magnitude band (Table 3), except for Prem players, who had lower peak angular accelerations (PAAs) for high HAE magnitudes (> 50 g). Forwards accounted for a higher percentage of HAE exposures in tackles and rucks in all grades except U13 (Fig. 5b, c). Most significantly, compared with backs, forwards had 1.67 times the risk (RR 1.669, CI 1.148–2.425; *p* = 0.007) for > 30 g events in rucks but did not carry a higher risk for > 30 g events in tackles (RR 0.996, CI 0.90–1.102; *p* = 0.935).

**Table 3:** Comparison of median peak angular acceleration (PAA) by peak linear acceleration (PLA) magnitude for match events (tackles, rucks, other) presented by playing grade

Age grade	PLA (g)	PAA (rads/s^2^)								
		Tackle area			Ruck area			Other		
		Count	Median	IQR	Count	Median	IQR	Count	Median	IQR
U13	5–10	188	746.44	(487.19)	40	666.14	(380.86)	15	626.17	(664.19)
	10–19	295	1210.60	(849.07)	90	1053.94	(775.67)	35	1304.80	(939.11)
	20–29	101	1951.84	(1266.26)	30	2076.77	(1443.37)	14	2370.71	(877.51)
	30–49	28	2594.66	(1547.43)	7	3389.08	(2701.02)	1	3188.37	0.00
	50 +	27	5566.28	(2134.74)	5	6675.72	(1711.52)	1	4932.04	0.00
U15	5–10	196	747.05	(372.65)	54	627.65	(357.97)	51	647.67	(534.27)
	10–19	580	1047.10	(693.21)	205	995.00	(563.14)	110	1258.35	(1276.56)
	20–29	229	1888.86	(1171.60)	70	1828.00	(1261.86)	33	2317.95	(1171.73)
	30–49	78	2761.08	(1692.96)	22	2440.20	(1653.39)	7	4514.77	(3162.63)
	50 +	50	4968.66	(2763.89)	21	3636.90	(2631.24)	6	3555.43	(2888.29)
U19	5–10	165	666.06	(385.86)	40	665.51	(241.81)	32	455.24	(447.87)
	10–19	470	1024.97	(601.82)	174	862.85	(610.86)	75	965.74	(625.55)
	20–29	149	1685.92	(933.24)	77	1659.02	(1050.56)	14	1415.38	(678.31)
	30–49	78	2721.21	(1104.66)	28	2213.46	(1256.40)	7	3140.48	(2284.46)
	50 +	61	4511.63	(2756.34)	18	3603.15	(2299.41)	5	4954.29	(2833.46)
Premier	5–10	116	635.78	(371.16)	35	575.06	(217.74)	50	516.80	(556.43)
	10–19	475	966.17	(540.97)	186	892.62	(564.32)	84	928.39	(622.58)
	20–29	157	1651.14	(825.03)	59	1489.24	(893.40)	21	2050.43	(889.29)
	30–49	60	2356.03	(1172.09)	18	2114.49	(1343.99)	6	3748.60	(1914.61)
	50 +	54	3945.17	(2929.91)	18	2992.87	(2341.45)	3	3567.56	(1028.08)

## Discussion

This study examines the head acceleration burden in community rugby over a 4-week playing period in playing grades from U13 to Prem men. Our results provide novel information on exposure to HAEs in community games, particularly concerning age and playing positions, with potential implications for training and match interventions to manage head impact burden in the sport.

### Weekly Exposure

The time spent exposed to rugby was proportionally similar across the grades, with the match and each training session representing approximately one-third of the total exposure time. Although the proportional exposure time was similar between the grades, HAE distribution was not. Matches accounted for 41% of the week's HAE exposure in the youngest players (U13), and this increased with each successive grade, with the most significant proportion of HAEs during matches (65% of all HAEs) in Prem men.

This finding is important in three ways. First, it shows that per hour of rugby, matches are more likely to cause HAEs than training sessions, in all age grades. Second, the increase in match contribution with age reflects the relative increase in match intensity that occurs naturally with playing experience and physical development. This is supported by findings that match injury incidence increases with playing level and age [32]. Third, it may reflect differences in training approaches as age and overall season length increases. While the in-season competition period is similar across age grades (3–4 months), the pre-season training/preparation period varies from one week in U13 players up to 10 weeks in U19 and Prem players. Because older players have longer pre-season preparation, they can dedicate more training time in-season to tactical preparation. Hence, older players dedicated one training session per week to contact and one to tactical preparation, which was reflected in the percentage difference in HAEs between T_1_ and T_2_ for U19 and Prem. In contrast, as younger players have an overall shorter pre-season, they have more of a contact focus in both weekly training sessions and spend more time developing technical proficiency for contact in both sessions [33]. This difference in the training approach would expose younger players to cumulatively more contact during training sessions; thus, HAEs are relatively more likely to occur during training than matches in these groups. This may have implications for how the sport advises coaches on training focus and contact loads.

For the following discussion point, it may be helpful to introduce some context for the range and magnitude of HAEs. To begin, our video verification revealed interquartile ranges associated with direct (5–26 g), indirect (5–18 g) and voluntary (5–12 g) HAE mechanisms (Supplemental Table 2, see ESM). From the wider literature, HAEs up to 6 g have been reported on roller coasters [34], up to 10 g have been reported for trampolining [35], 10–19 g have been reported in women’s artistic gymnastics [36] and 20–30 g have been reported in automobile testing for rear impact collisions [37]. The automobile study noted that concussion risk in rear impact collisions of this magnitude was negligible [34], while recent iMG studies of American Football players identified HAEs associated with diagnosed concussion events that ranged between 40–150 g [11, 38]. With this context in mind, we would propose that HAEs < 29 g are *low risk* while events > 30 g carry higher potential for injury.

In our community rugby cohort, HAEs of 10–29 g occurred at 6–8 times the rate of 30–60 g events. The IR for > 30 g events ranged from 1/week in our youngest cohort to 2.2/week in the U19s. It is important to note that IRs represent an average per-player exposure and that HAEs are not necessarily spread evenly among the population [39]. For example, U19 and Prem had similar IRs for 30–60 g match events, yet 63% of U19 players were exposed to at least one 30–60 g event, compared with 58% of Prem players (Table 2). Therefore, the relative risk of experiencing a high-magnitude HAE is 1.36 times higher for an U19 player than for the rest of the playing population. This finding might also suggest that some Prem players experience a larger proportion of 30–60 g events relative to their cohort. Examining the techniques and behaviours of these ‘higher risk’ players may be warranted to determine the HAE aetiology.

The fact that school-based U19 players are more likely to experience at least one 30–60 g event during match play is consistent with previous studies that highlighted an increase in match injury rates in this population [40]. Our U19 cohort were recruited from ‘1st XVs’, which is the top tier for schoolboy rugby. Top tier schoolboy teams also have a higher level of professionalism and physical preparation, and attract a high level of community interest in spectator attendance and fanfare, which can significantly impact playing behaviour, leading to higher contact intensity, poorer decision making, and reduced focus on technique [41–43]. Larger urban schools may have paid professional coaches, dedicated strength and conditioning programmes, links to professional teams, and televised interschool matches [44]. The level of professionalism improves skill development and readiness to play but may have a negative consequence regarding game intensity [45]. Further examination of match events and mechanisms that lead to higher HAEs in U19 school-based players is required.

### Exposure by Playing Position and Contact Type

Consistent with the previous literature, the weekly HAE exposure rate was higher for forwards than for backs [8, 21, 46–49]. This is likely the result of their greater exposure to contact events, since forwards typically make more tackles and carries, and are involved in more rucks than backs, and training activities will reflect these match demands.

The positional difference was largest at low magnitudes in older players for match exposure, with the position and session-type gap narrowing with decreasing age. Consequently, U13s had the opposite position incidence profile, with backs experiencing more HAEs in training sessions at lower magnitudes. The U13 backs also had a higher proportion of HAEs in the tackle (56%) and rucks (54%) than the U13 forwards. This discrepancy could reflect the lack of specialisation in positional play in the U13s, where player size, development, and skill level may be more significant factors for contact events [50]. The lack of positional specialisation could also explain the greater training exposure in U13s. In higher grades, training sessions involve ‘units’ where the drills will be specifically tailored to the match requirements of that position group. The lack of specialisation and increased attention on basic skills in U13s is the likely reason for the similar HAE incidence in U13 forwards and backs.

Apart from U13s, all grades had higher weekly numbers of 10–29 g events during match play than during training. During match play, the tackle was responsible for 66–75% of HAE exposure, with forwards contributing the highest proportion of those events (44–65%). Surprisingly, we found no difference in the prevalence of high-magnitude (30–60 g) tackle-related HAEs in forwards compared with backs. This finding may be incongruent with the current literature, which suggests that while forwards perform more 'heavy and severe' impacts than backs [51], backs have higher risk for tackle concussion injury due to larger tackle entry velocities [3]. The lack of positional differences in our sample could reflect a difference in player skill or athleticism at the community level compared with elite/professional games. However, further exploration of tackle-related HAEs in community games is necessary to provide deeper insights and context for these observations.

The ruck area was the second largest contributor (18–24%) to match HAEs, with forwards accounting for 46–78% of the burden depending on age grade. Moreover, forwards were significantly more likely than backs to experience a 30–60 g event in a ruck. It is well known that tackles and rucks carry the highest risk of injury and that players with more involvement in these events would likely also carry a higher risk of injury [3, 52]. Thus, our findings may suggest that in the community game, the back-row forwards carry a higher concussion risk in ruck events because these players are 'typically' the first to arrive at the breakdown [53]. There is some evidence in the professional and community adult grades supporting this supposition [53, 54]. However, future work will provide richer insights into the ruck and positional differences, to further elucidate whether the risk is due to player technique, player behaviour, rule enforcement, or intensity control.

### Limitations

This study had several limitations. First, iMG fit quality may have differed between the grades. While smaller players were offered smaller-sized iMGs with a lower overall volume of material, obtaining optimal fit may still be difficult because younger players were more likely to have a narrow dental arch, triangular bite, and missing or misaligned teeth. Poorer iMG fit may lead to a higher volume of false and voluntary triggers of the iMG. We did all we could to mitigate the fit effect by having qualified dentists perform the iMG fitting and tracking the iMG-tooth displacement from the proximity sensor, which gave us a quality-of-fit score. Secondly, we could not fully video verify all training HAEs owing to poor video quality. Therefore, we relied on our impressions from the waveform data to distinguish between voluntary versus direct/indirect events. Furthermore, we could only confirm tackle- and ruck-related HAEs from the match sessions. We are currently working on optimisation techniques to be used in the future to elucidate tackle- and ruck-related events in the training sessions. Thirdly, we did not follow teams over the entire season because of time constraints and the large cohort of athletes we tracked. Instead, we chose to observe a minimum of 4 weeks of exposure time, meaning at least four matches and eight training sessions per team. The length of the observation period was controlled, but staggered by grade over the season. This means that some grades might have been captured at the beginning of the season and others near the end. Seasonal differences could affect the intensity of play or training, although the mixed-model procedure adopted in the analysis should accommodate these variations. Finally, our current study did not include information on concussions or other types of injuries, which may have important implications in injury identification, prevention and management. This will be the focus of future research.

## Conclusions/Implications

The findings of this study have important implications for community rugby, particularly with respect to age, position, and session exposure. The results suggest that playing grade/age and position significantly influence HAE exposure incidence, with forwards experiencing the highest incidence, particularly during match play in tackle and ruck areas. Consequently, it may be prudent for community rugby coaches and referees to focus attention on tackle and ruck techniques, particularly in the U19 grades. A focus on managing the intensity of match play at higher grades in the school environment may also warrant further investigation. The study also found that the contribution of training sessions to weekly HAE exposure drastically diminished with age. This has important implications for how the sport guides training load, particularly for younger players where skill acquisition is still in the associative phase and coaching of close contact skills often takes priority over the strategy coaching that is often seen in older players. Youth community coaches may need to develop alternative approaches to coaching contact skills without full contact load.

This study provides new information on the exposure to HAEs in community rugby and marks the beginning of our investigation of this cohort. Clearly, there is a need for ongoing research to understand the mechanisms that lead to HAEs and develop targeted injury prevention strategies for high-risk players.

### Supplementary Information

Below is the link to the electronic supplementary material.

Supplementary file1 (DOCX 1980 KB)

## Declarations

### Data availability statement

Data are available on reasonable request to the corresponding author.

### Patient and public involvement

Patients and/or the public were not involved in the design, conduct, reporting, or dissemination plans of this research.

### Equity, diversity, and inclusion statement

Our study was on male community rugby players in New Zealand. Our study included men and boys playing rugby at grassroots levels, mainly from the Otago region, and represent various cultural (NZ European, Māori, Pasifika and other Asian nations) and socioeconomic backgrounds. The research team included four women and three men (first and second authors are women). The authors’ disciplines include exercise science, physiotherapy, mechanical engineering, biomedical sciences and sports medicine.
