# Supplementary material for: Head Acceleration Events in Male Community Rugby Players: An Observational Cohort Study across Four Playing Grades, from Under-13 to Senior Men
Source: Sports Med. 2023 Sep 7;54(2):517–30. doi: 10.1007/s40279-023-01923-z (PMC10933157; doi:10.1007/s40279-023-01923-z)
Supplement: Supplementary file 1 — Supplementary file1 (DOCX 1980 KB) [file 40279_2023_1923_MOESM1_ESM.docx]

Supplementary Materials

Head Acceleration Event Exposure in Male Community Rugby Players: An observational cohort study across four playing grades from Under-13 to Senior Men.

Melanie D Bussey^1^, Danielle Salmon^2^, Janelle Romanchuk^1,2^, Bridie Nanai^1^, Peter Davidson^1^, Ross Tucker ^3,4^, Eanna Falvey^4,5^

1 School of Physical Education, Sports and Exercise Sciences, University of Otago;2 New Zealand Rugby;3 Institute of Sport and Exercise Medicine, University of Stellenbosch; 4 World Rugby, Dublin, Ireland; 5 School of Medicine & Health, University College Cork, Ireland

Associate Professor Melanie Bussey (corresponding author) [melanie.bussey@otago.ac.nz](mailto:melanie.bussey@otago.ac.nz)

Dr Peter Davidson [peter.davidson@otago.ac.nz](mailto:peter.davidson@otago.ac.nz)

Dr Danielle Salmon danielle.salmon@worldrugby.org

Janelle Romanchuk [janelle.romanchuk@postgrad.otago.ac.nz](mailto:janelle.romanchuk@postgrad.otago.ac.nz)

Bridie Nanai [bridie.nanai@otago.ac.nz](mailto:bridie.nanai@otago.ac.nz)

Professor Ross Tucker [ross@sportsscientists.com](mailto:ross@sportsscientists.com)

Professor Éanna Falvey [Eanna.Falvey@worldrugby.org](mailto:Eanna.Falvey@worldrugby.org)

Word count 3446; Tables 3; Figures 5; Supplementary Tables 1; Supplementary Figures 3


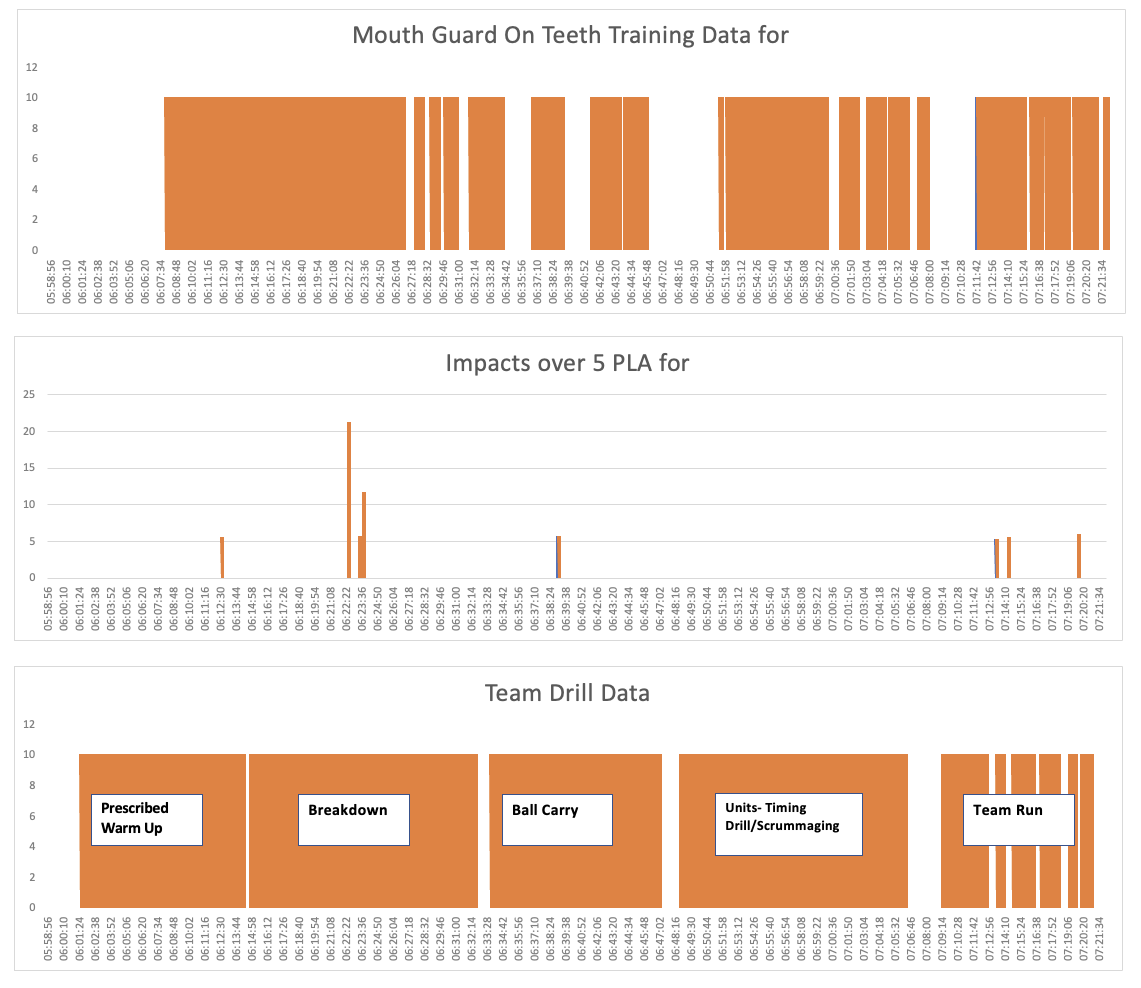


Figure 1 Example of temporospatial data windowing for training events in a single representative player. The top figure is proximity data from IMG, green blocks represent 'on-teeth' time, black blocks represent 'off-teeth' time. Middle figure showing true HAE events > 5 g recorded for player. Figure in the bottom shows temporal blocks for each training drill in which the player participated while wearing the IMG.


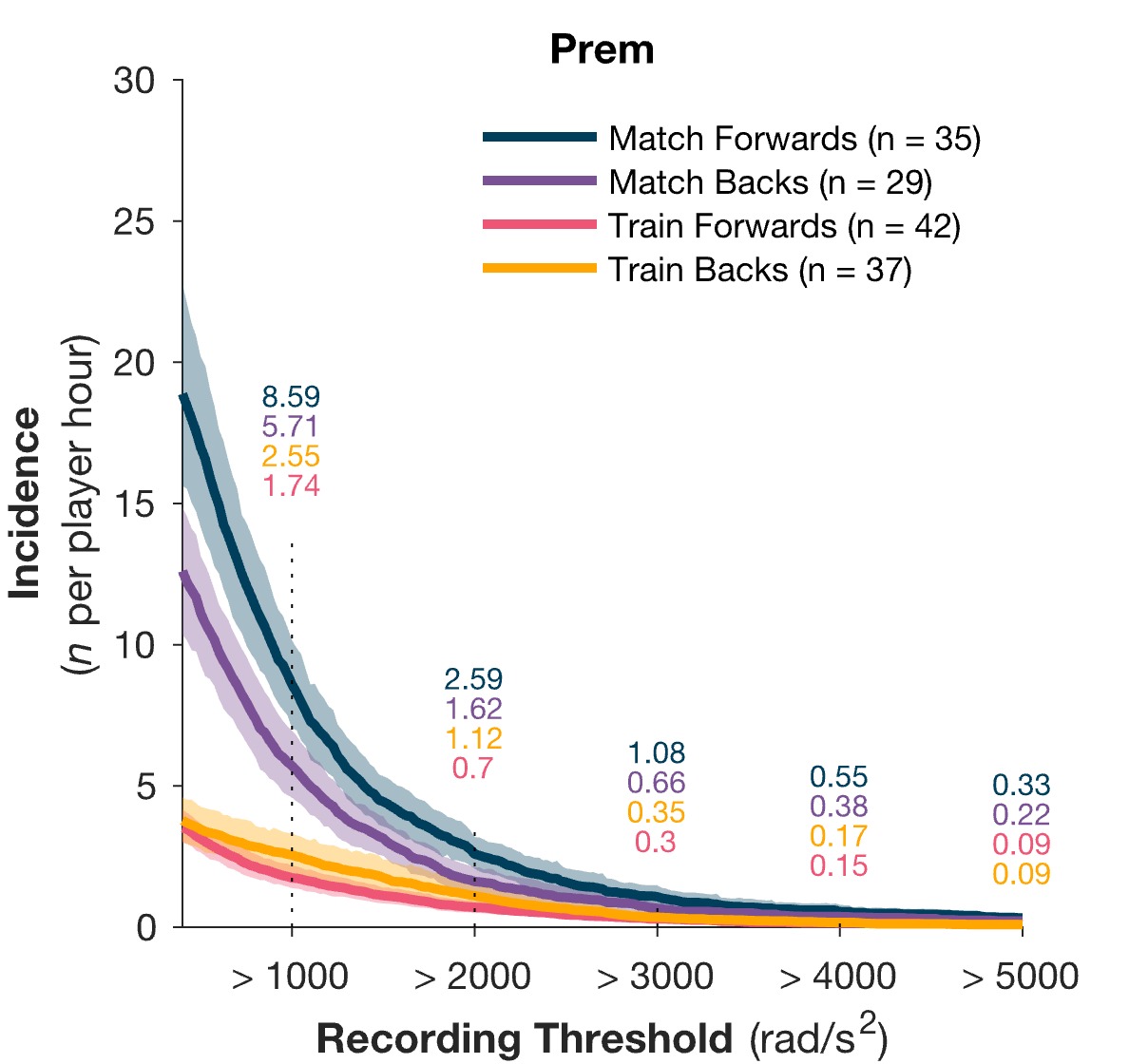

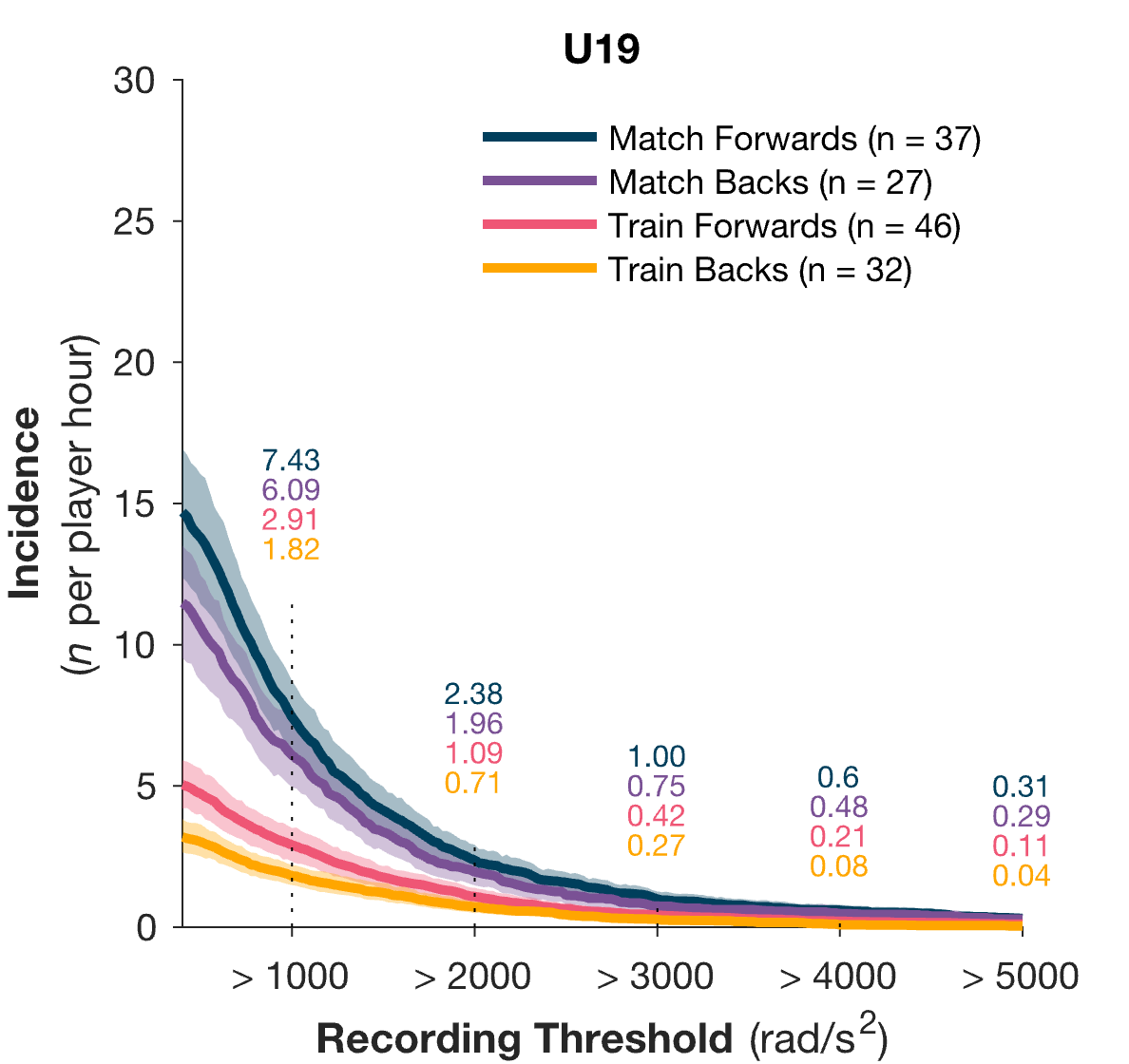

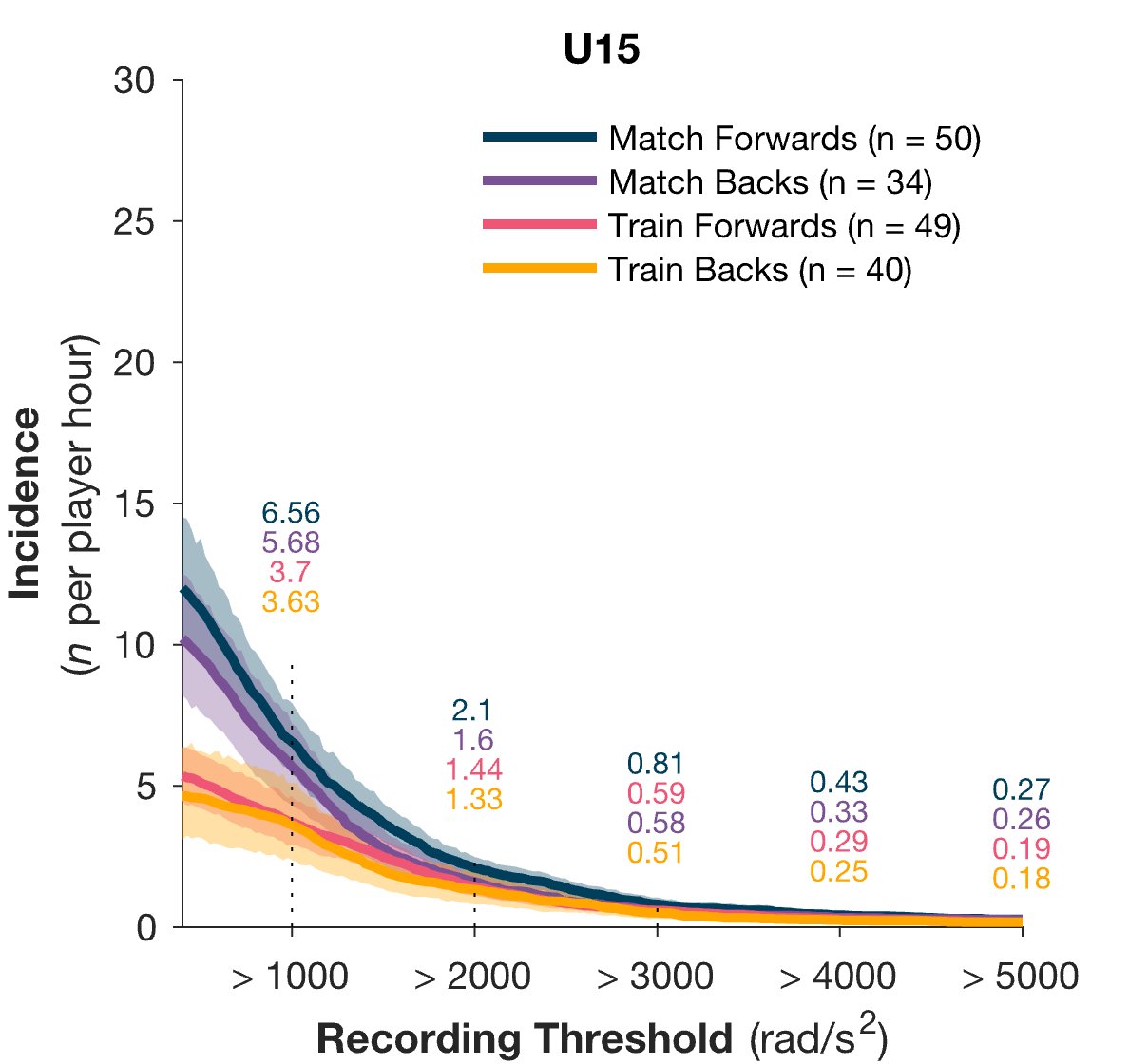

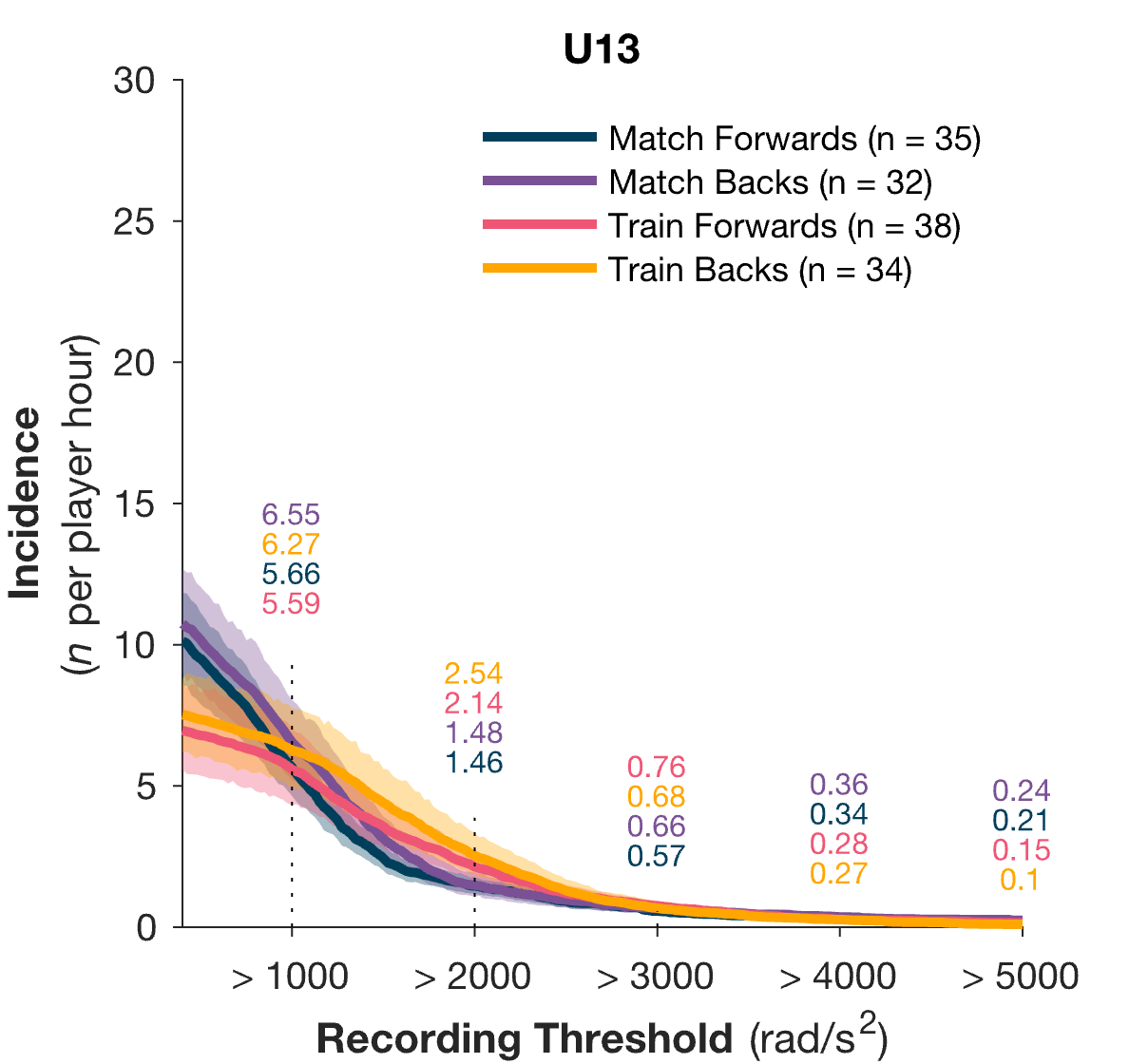


Figure 2: HAE incidence rate per player hour for match forwards (blue), match backs (purple), training forwards (pink) and training backs (yellow) across the four playing grades: Premier, U19, U15 and U13, a-d, respectively. The solid line represents the mean incidence rate and shading represents the 95% confidence interval. HAE incident rates per PAA threshold band (1000 – 5000 rads/s^2) are indicated by the numbers above each vertical line.


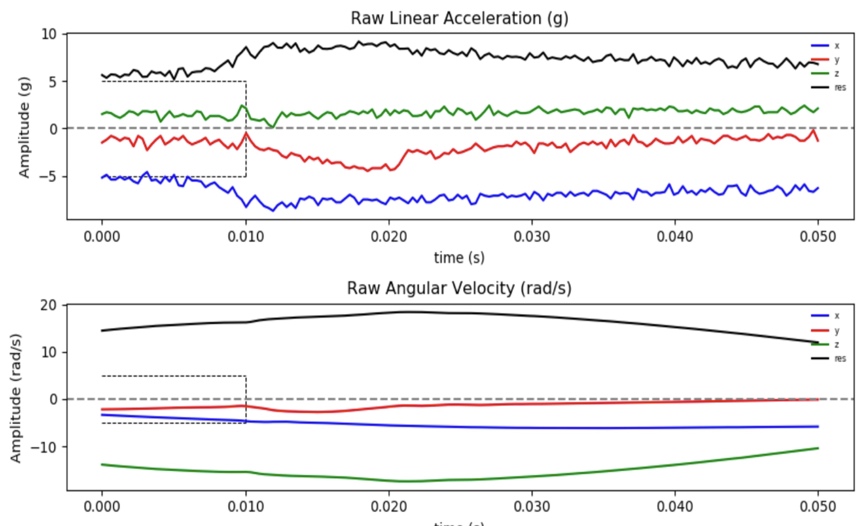


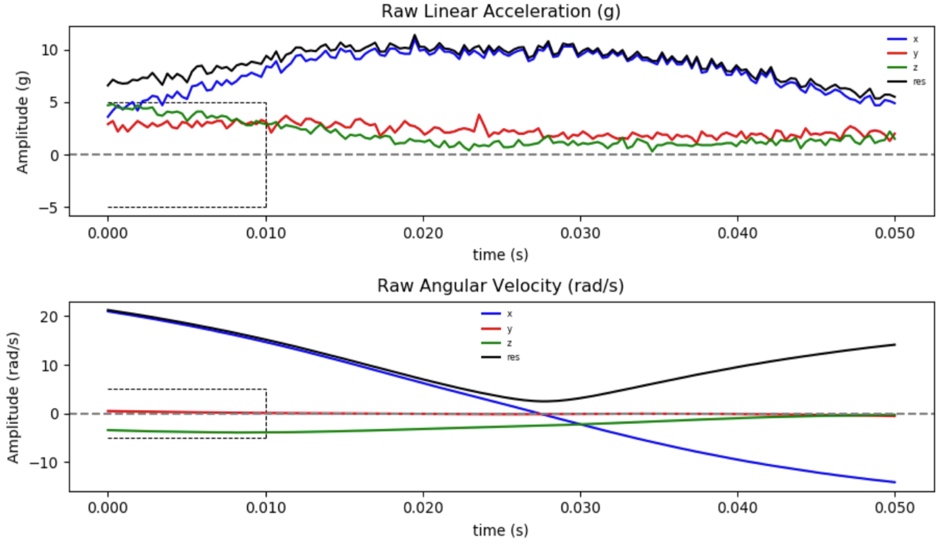


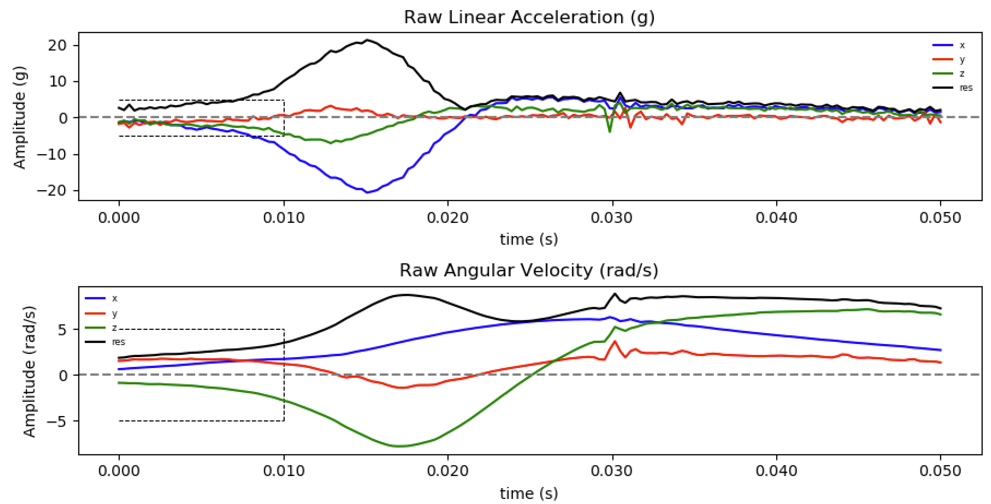


Figure 3: Example of HAE raw waveform examination. The top left figure shows a fault identified in the gyroscope. The top right figure shows a fault identified in the accelerometer. The bottom figure presents an example of what would be expected as a typical high-quality signal.

| Playing  Grade | Type of  HAE | Forwards | | | | | Backs | | | | | | | |
| --- | --- | --- | --- | --- | --- | --- | --- | --- | --- | --- | --- | --- | --- | --- |
|  |  | Count | PLA (g)  Median | IQR | PAA (rads/s^2)  Median | IQR | Count | PLA (g)  Median | IQR | | PAA (rads/s^2)  Median | | | IQR |
| U13 | Training | 1027 | 12.7 | (8.77) | 1511.39 | (1140.80) | 1214 | 13.4 | | (7.73) | | 1723.98 | (1067.50) | |
|  | Match | 659 | 10.28 | (6.98) | 1117.3 | (749.42) | 831 | 10.4 | | (6.97) | | 1253.89 | (793.87) | |
|  | Direct | 300 | 13.71 | (10.39) | 1172.51 | (1151.35) | 365 | 13.86 | | (10.35) | | 1276.72 | (1054.99) | |
|  | Indirect | 61 | 11.15 | (6.73) | 1135.19 | (1095.63) | 77 | 11.86 | | (8.84) | | 1403.42 | (1592.58) | |
|  | Unclear | 165 | 8.1 | (3.70) | 975.52 | (515.56) | 200 | 7.9 | | (3.30) | | 1187.76 | (611.55) | |
|  | Vol | 133 | 8.98 | (3.03) | 1157.19 | (514.23) | 189 | 9.07 | | (2.75) | | 1281.84 | (440.23) | |
| U15 | Training | 1067 | 12.24 | (9.15) | 1331.01 | (1314.31) | 552 | 12.12 | | (8.78) | | 1440.44 | (1037.88) | |
|  | Match | 1903 | 11.93 | (9.88) | 1078.99 | (1020.60) | 962 | 10.89 | | (8.32) | | 1089.37 | (851.68) | |
|  | Direct | 1074 | 15.49 | (10.87) | 1195.71 | (1123.95) | 415 | 15.15 | | (11.21) | | 1204.09 | (1000.67) | |
|  | Indirect | 126 | 12.51 | (7.91) | 1212.1 | (1729.77) | 97 | 10.25 | | (6.35) | | 1061.69 | (1097.26) | |
|  | Unclear | 269 | 7.7 | (1.80) | 892.89 | (586.49) | 133 | 7.4 | | (1.70) | | 974.49 | (585.05) | |
|  | Vol | 434 | 8.99 | (4.48) | 825.84 | (1034.19) | 317 | 9.51 | | (4.32) | | 1023.45 | (812.11) | |
| U19 | Training | 1043 | 12.68 | (9.64) | 1144.99 | (1197.06) | 372 | 12.76 | | (9.31) | | 1029.13 | (1101.73) | |
|  | Match | 1433 | 12.33 | (9.33) | 971.14 | (925.53) | 774 | 12.07 | | (9.26) | | 983.07 | (910.89) | |
|  | Direct | 816 | 15.31 | (11.58) | 1145.26 | (1050.83) | 388 | 16.21 | | (11.53) | | 1140.79 | (1063.31) | |
|  | Indirect | 86 | 11.65 | (7.30) | 846.33 | (1084.30) | 78 | 9.86 | | (6.17) | | 863.43 | (688.02) | |
|  | Unclear | 290 | 10.00 | (5.70) | 794.8 | (636.28) | 128 | 10.45 | | (4.60) | | 887.7 | (617.18) | |
|  | Vol | 241 | 9.00 | (2.59) | 668.17 | (689.35) | 180 | 8.86 | | (3.90) | | 685.93 | (970.98) | |
| Prem | Training | 590 | 12.10 | (10.31) | 929.4 | (1116.26) | 580 | 12.37 | | (9.20) | | 1272.77 | (1398.64) | |
|  | Match | 1558 | 11.78 | (9.33) | 877.15 | (817.75) | 1086 | 9.93 | | (7.42) | | 806.36 | (828.88) | |
|  | Direct | 805 | 15.79 | (11.19) | 1099.45 | (989.59) | 371 | 15.16 | | (10.06) | | 1060.16 | (936.90) | |
|  | Indirect | 81 | 10.17 | (5.31) | 752.86 | (751.07) | 86 | 10.79 | | (7.32) | | 886.11 | (710.89) | |
|  | Unclear | 169 | 11.10 | (7.70) | 857.56 | (729.04) | 98 | 9.1 | | (5.20) | | 893 | (726.85) | |
|  | Vol | 503 | 8.05 | (2.45) | 599.06 | (534.59) | 531 | 8.14 | | (2.93) | | 562.16 | (770.55) | |

Table 1: Description of video verified HAEs by mechanism, playing grade and position.
